# Predictors of contralateral breast cancer in BRCA1 and BRCA2 mutation carriers

**DOI:** 10.1038/bjc.2011.120

**Published:** 2011-04-12

**Authors:** K Metcalfe, S Gershman, H T Lynch, P Ghadirian, N Tung, C Kim-Sing, O I Olopade, S Domchek, J McLennan, A Eisen, W D Foulkes, B Rosen, P Sun, S A Narod

**Affiliations:** 1 Lawrence S Bloomberg Faculty of Nursing, University of Toronto, Toronto, Canada; 2 Women's College Research Institute, 790 Bay Street, Room 750, Toronto, Ontario, Canada M5G 1N8; 3 Department of Preventive Medicine and Public Health, Creighton University School of Medicine, Omaha, NE, USA; 4 Epidemiology Research Unit, Centre Hospitalier de Université de Montreal (CHUM), Montreal, Quebec, Canada; 5 Beth Israel Deaconess Medical Center, Boston, MA, USA; 6 BC Cancer Agency, Vancouver, British Columbia; Canada; 7 Department of Medicine, University of Chicago, Chicago, IL, USA; 8 Departments of Medicine and Genetics, Department of Hematology/Oncology, University of Pennsylvania, PA, USA; 9 Cancer Risk Program, UCSF Comprehensive Cancer Center, San Francisco, CA, USA; 10 Toronto Sunnybrook Regional Cancer Center, Toronto, Ontario, Canada; 11 Program in Cancer Genetics, McGill University, Montreal, Quebec, Canada; 12 University Health Network, Toronto, Ontario, Canada

**Keywords:** breast cancer, BRCA1, BRCA2, contralateral breast cancer

## Abstract

**Purpose:**

The objective of this study was to estimate the risk of contralateral breast cancer in BRCA1 and BRCA2 carriers; and measure the extent to which host, family history, and cancer treatment-related factors modify the risk.

**Patients and methods:**

Patients were 810 women, with stage I or II breast cancer, for whom a BRCA1 or BRCA2 mutation had been identified in the family. Patients were followed from the initial diagnosis of cancer until contralateral mastectomy, contralateral breast cancer, death, or last follow-up.

**Results:**

Overall, 149 subjects (18.4%) developed a contralateral breast cancer. The 15-year actuarial risk of contralateral breast cancer was 36.1% for women with a BRCA1 mutation and was 28.5% for women with a BRCA2 mutation. Women younger than 50 years of age at the time of breast cancer diagnosis were significantly more likely to develop a contralateral breast cancer at 15 years, compared with those older than 50 years (37.6 *vs* 16.8% *P*=0.003). Women aged <50 years with two or more first-degree relatives with early-onset breast cancer were at high risk of contralateral breast cancer, compared with women with fewer, or no first-degree relatives with breast cancer (50 *vs* 36% *P*=0.005). The risk of contralateral breast cancer was reduced with oophorectomy (RR 0.47; 95% CI 0.30–0.76; *P*=0.002).

**Conclusion:**

The risk of contralateral breast cancer risk in BRCA mutation carriers declines with the age of diagnosis and increases with the number of first-degree relatives affected with breast cancer. Oophorectomy reduces the risk of contralateral breast cancer in young women with a BRCA mutation.

Women who carry a germline mutation in either the *BRCA1* or the *BRCA2* gene face a high lifetime risk of breast cancer (Ford *et al*, 1998) and, once diagnosed with breast cancer, face a high risk of second primary cancer in the contralateral breast (Robson *et al*, 1998; Verhoog *et al*, 1998, 2000; Haffty *et al*, 2002; Metcalfe *et al*, 2004; Brekelmans *et al*, 2007; Graeser *et al*, 2009). The 10-year contralateral breast cancer risk has been estimated at between 13 and 40% for women with a BRCA mutation. It is important to identify factors, which predict the risk of contralateral breast cancer in this group of high-risk women to provide optimum genetic counselling and to inform treatment decisions.

We have recently reported that a family history of cancer influences breast cancer risk in women with a BRCA1 or BRCA2 mutation (Metcalfe *et al*, 2010). Among women with a BRCA1 mutation, the risk of breast cancer increased by 1.2-fold for each first-degree relative with breast cancer before age 50 years (HR=1.21; 95% CI 0.94–1.57), and among women with a BRCA2 mutation, the risk of breast cancer increased by 1.7-fold for each first-degree relative with breast cancer (HR=1.65; 95% CI 1.00–2.71). It is unclear whether family history of cancer also influences the risk of contralateral breast cancer in these high-risk women.

Although there have been several studies that estimate the risk of contralateral breast cancer in women with a BRCA1 or BRCA2 mutation, there has been little research on the predictors of contralateral breast cancer risk. In 2004, we reported on the experience of 336 women with a BRCA mutation. Since this report, we have extended the study sample from 336 patients to 810 patients, and we have extended the mean follow-up period from 9.2 to 11.1 years. In addition, we have collected detailed information on the family histories of the breast cancer patients. Using this cohort, we estimate the contralateral breast cancer risks in women with a BRCA1 or BRCA2 mutation, and we measure the extent to which host factors, family history, and cancer treatments modify the risk.

## Patients and Methods

To identify study subjects, the pedigrees of BRCA families who were received genetic counselling at the 10 participating cancer genetics clinics were reviewed. A family was considered to be eligible for the study when a BRCA1 or a BRCA2 mutation was documented in the family and at least one case of invasive breast cancer was recorded. Eligible study subjects included all women from these families who were diagnosed with Stage I or Stage II breast cancer at 65 years of age or below, between 1975 and 2008. Living and deceased women were eligible, but those with a previous diagnosis of cancer (including breast cancer) or those who resided outside of North America were excluded. It was not necessary to be a proven carrier of the mutation found in the family to be included in the study; however, affected women who were known to be non-carriers were excluded. All study procedures were approved by the Institutional Review Boards at each of the participating centres.

We identified a total of 1866 breast cancer cases in 615 families. Of the total 1866 cases of breast cancer, 417 women were excluded because the date of diagnosis indicated on the pedigree was before 1975, and 70 women were excluded because the age of diagnosis was above 65 years. An additional 29 women were known not to carry the familial mutation and were therefore excluded. In total, 19 women were excluded because they had a diagnosis of other cancer before breast cancer, and 26 women were excluded because they were treated outside of North America.

We were able to obtain identifying information for 993 of the remaining 1305 women (76%). An attempt was made to contact each of these or her next-of-kin to obtain permission to review the medical records. Overall, 19 women (or their next of kin) refused to provide consent for the release of the medical records. The medical record was requested from the hospital where treatment was received for the remaining 974 women. In 76 cases, the hospital was not able to locate the record or did not forward the requested documents. The medical record was obtained for the remaining 898 women (92%).

After review of the medical records, an additional 52 women were excluded. Of these, 32 women were excluded because tumour stage was greater than two; 18 women were excluded because the tumour was non-invasive (DCIS or LCIS), and 2 women were excluded because they refused treatment. The remaining 846 women were included in the analysis. In summary, of the 1866 breast cancers, 613 were ineligible and we were able to enroll 846 of the remaining 1253 eligible cases (68%). 177 of the 846 women were deceased (20.9%).

### Study protocol

The medical treatment records and pathology documents were reviewed. We established whether the tumour was unilateral or bilateral at initial diagnosis. We recorded tumour size (in cm), nodal status (positive/negative), and tumour grade (I to III). Where possible, we abstracted information on both mitotic and nuclear grade. Status of ER was recorded as positive, negative, equivocal, or unknown. We recorded the use of chemotherapy (yes/no), tamoxifen (yes/no), and radiotherapy (yes/no). We established whether or not the patient had undergone a bilateral oophorectomy, and if so, the date of the operation. In some cases, the contralateral mastectomy and/or oophorectomy were performed several years after the initial surgical treatment; the dates of these late treatments were recorded. We reviewed the dates of diagnosis of all contralateral breast cancers reported in the cohort. Only invasive contralateral cancers were included. No woman was diagnosed with contralateral breast cancer after she was diagnosed with distal metastases. Information on family history was collected on the patients by review of the pedigree at the time of ascertainment. Each patient was classified according to the number of documented first-degree relatives with early-onset breast cancer (diagnosed at or below age 50 years) as zero, one, or two or more affected relatives.

### Statistical analysis

A survival analysis was performed. We considered the woman to be at risk for contralateral breast cancer from the date of the first surgical procedure to the first of contralateral breast cancer, contralateral mastectomy, death from breast or ovarian cancer, death from another cause, or the date of last follow-up. The date of the last follow-up was defined as date of last contact or death. Survival curves were constructed and compared for subgroups of women defined by age (<40; 41–50 or >50 years), family history (number of first-degree relatives with breast cancer), and by mutation status (BRCA1 *vs* BRCA2). We also compared the risk of contralateral breast cancer for subgroups defined by each of the four treatments individually (i.e., tamoxifen, radiotherapy, oophorectomy, chemotherapy). Hazard ratios were estimated using the Cox proportional hazards model, implemented in SAS (SAS Institute Inc., Cary, NC, USA). In these analyses, oophorectomy was treated as a time-dependent variable. The hazard ratios were adjusted for age at first cancer diagnosis, mutation status (BRCA1 *vs* BRCA2), family history, and other treatments received.

## Results

A total of 846 women with complete medical information were identified, and of these, 810 women with stage I or II breast cancer and an intact contralateral breast were included in the current analysis. There were 498 women from families with a BRCA1 mutation and 300 women from families with a BRCA2 mutation. Overall, 12 women carried a mutation in both genes. In all, 87.8% patients had the BRCA mutation confirmed by sequencing and the remaining 12.2% had not had genetic testing but were from a family with a known BRCA mutation. The characteristics of the 810 women (including demographics, tumour characteristics, and treatments) are presented in Table 1.

Patients were diagnosed between 1975 and 2008. The subjects were followed for a mean of 11.1 years (range 0.1–32.9 years). In all, 78.8% of the subjects were alive at the time of last follow-up. In total, 149 subjects (18.4%) were diagnosed with a contralateral breast cancer. All contralateral cancers were confirmed with the medical record. The mean time interval period between the diagnosis of the first breast cancer and the diagnosis of the contralateral breast cancer was 5.7 years (range 0.2–15 years).

**Figure 1:**
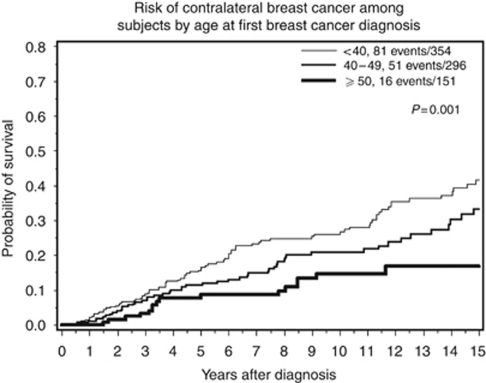
Risk of contralateral breast cancer among subjects by age at first breast cancer diagnosis. The end points for the three survival curves are 0.416, 0.330, and 0.168, respectively.

In the entire sample, the 5-year actuarial risk of contralateral breast cancer was 13.1% (95% CI 10.3–15.9%), the 10-year risk was 22.0% (95% CI 19.2–26.8%), and the 15-year risk was 33.8% (95% CI 28.6–39.0%). The annual risk was 2.1%. The 5-, 10-, and 15-year cumulative risks of contralateral breast cancer were estimated for each age group and by mutation status (Table 2). The risk of contralateral breast cancer was estimated for patient subgroups defined by age group (Figure 1), gene (*BRCA1 vs BRCA2*), number of affected first-degree relatives, and by treatment received (surgery, chemotherapy, tamoxifen, radiotherapy, and ovarian ablation). The univariate and multivariate hazard ratios associated with each of these factors are presented in Table 3. Women who were aged 50 years or above at the time of breast cancer diagnosed experienced a significantly reduced risk of contralateral breast cancer, compared with those diagnosed younger than age 40 years (RR 0.47; 95% CI 0.47−0.82; *P*=0.008).

The risk of contralateral breast cancer also depended on the family history of the patient. The effect of family history was only present for women whose initial breast cancer was diagnosed at age 49 years or below. For these women, the 15-year risks of contralateral breast cancer were estimated to be 33.4, 39.1, and 49.7% for women with zero, one, and two or more first-degree relatives with breast cancer diagnosed under age 50 years.

Out of 810, 489 women (60.4%) underwent bilateral oophorectomy; 40 women before breast cancer diagnosis, 354 women within the year following breast cancer surgery, and 75 women at a later date (20 dates missing). Subjects with an oophorectomy had a significantly lower risk of contralateral breast cancer, compared with women without an oophorectomy (RR 0.48; 95% CI 0.27−0.82; *P*=0.002) (Table 3). The risk reduction associated with oophorectomy was significant for women diagnosed with the initial breast cancer before the age of 50 years (RR 0.39; 95% CI 0.23–0.67; *P*=0.0006), but not for those with age 50 years or older (RR 0.90; 95% CI 0.30–2.64; *P*=0.84) (Table 4). For women with a BRCA1 mutation, oophorectomy was associated with a significant reduction in the risk of contralateral breast cancer in BRCA1 carriers (RR 0.48; 95% CI 0.27–0.84; *P*=0.01) (Table 5). Oophorectomy was associated with a 51% reduction in contralateral breast cancer risk in BRCA2 carriers, but this was not statistically significant (*P*=0.11) (Table 5). Among women with two intact ovaries and who were under age 50 years at first diagnosis, the 15-year cumulative incidence of contralateral breast cancer was 58%. In addition, if a woman in this subgroup also had two or more first-degree relatives with breast cancer, the 15-year risk was 68%.

Neither radiotherapy nor chemotherapy was associated with a statistically significant reduction in the risk of contralateral breast cancer. Tamoxifen use was associated with a reduced risk of contralateral breast cancer risk in BRCA1 carriers in the univariate analysis (RR 0.55; 95% CI 0.31–0.94; *P*=0.03); but not after adjustment for age of diagnosis and the other treatments (Table 5). Tamoxifen was not associated with a reduction in risk in BRCA2 carriers.

## Discussion

Women with BRCA mutations have an extremely high lifetime risk of developing breast cancer. In addition, the risk of contralateral breast cancer is also elevated. In the current expanded cohort, we estimate the 10-year contralateral breast cancer risks to be 24% for BRCA1 carriers and 19% for BRCA2 carriers. These risks are comparable to those in the report of Brekelmans *et al* (2007), which included 262 women with a BRCA mutation and comparable to those of (Graeser *et al*, 2009). In the latter study, the 10-year contralateral breast cancer risk was 23%. However, of the 1042 women included in the Graeser *et al* (2009), only 17% had a documented BRCA mutation. Some of the women included in the cohort may have had sporadic breast cancer in which the risk of contralateral breast cancer would have been lower. In our study, 88% of the women had their mutation status confirmed, and we excluded known sporadic cases. In addition, in the study by Graeser *et al* (2009), cancers in subjects were confirmed by medical records for 45% of the women, compared with 100% of the cancers in the present study.

Recently, Malone and colleagues (2010) compared contralateral breast cancer risks in women with and without BRCA mutations (Malone *et al*, 2010). They included 181 women with BRCA mutations and observed a four-fold increased risk of contralateral breast cancer in women with a BRCA mutation compared with those without. This equated to a 21% risk of contralateral breast cancer at 10 years in BRCA carriers. These results support our observations, and demonstrate the importance of knowing BRCA status at time of initial breast cancer diagnosis when making breast cancer treatment decisions.

It is also important to recognise the modifiers of contralateral breast cancer in women with BRCA mutations to effectively counsel women on their personal risk of contralateral breast cancer and how treatments may modify this risk. To our knowledge, ours is the first study that has reported on the influence of family history of cancer on contralateral breast cancer risk in women with a BRCA1 or BRCA2 mutation. Among BRCA1 carriers, each first-degree relative affected with breast cancer before 50 years of age was associated with a 40% increase in the risk of contralateral breast cancer. Although this observation has never been described previously, we have shown before that family history of cancer influences a woman's risk of a first primary breast cancer, most notably in women with a BRCA1 mutation (Metcalfe *et al*, 2010).

We also observed differences in the risks of contralateral breast cancer in women with a BRCA mutation according to the age at first breast cancer diagnosis. Women diagnosed with breast cancer younger than 40 years of age had a 15-year contralateral breast cancer risk of 42% (annual risk 2.8%) compared with 19% risk for women over the age of 50 years at time of diagnosis (annual risk 1.3%). These findings are consistent with those of Graeser *et al* (2009). In their study of relatives of BRCA1 mutation carriers, they reported a 25-year contralateral breast cancer risk of 63% for women younger than 40 years of age at time of first breast cancer (annual risk 2.5%) compared with 20% for those older than age 50 years (annual risk 0.8%) (Graeser *et al*, 2009).

In the current study, we did not observe a protective effect against contralateral breast cancer associated with chemotherapy use (RR 0.97; *P*=0.9) in the entire study population or in any subgroup. This finding contrasts with that of Reding *et al* (2010), which was based on 181 women, with a BRCA mutation, who were alive at the time of the study interview (Reding *et al*, 2010). These authors reported a 50% reduction in contralateral breast cancer associated with chemotherapy (*P*=0.04). In our study, we also included deceased cases to eliminate the potential for survival bias.

The strongest predictor of contralateral breast cancer in women with a BRCA mutation in this study was oophorectomy. This effect was observed in women who were diagnosed with their initial breast cancer under the age of 50 years (60% reduction in risk) and was significant for those with a BRCA1 mutation (53% reduction in risk). A recent meta-analysis was published of eight studies that estimated the risk of first primary breast cancer in BRCA1/2 mutation carriers who were treated with prophylactic oophorectomy relative to carriers who had intact ovaries (Rebbeck *et al*, 2009). The study included three studies that examined the breast cancer risk reduction in BRCA2 carriers specifically. Although two of the studies reported no significant breast cancer risk reduction associated with prophylactic oophorectomy in BRCA2 carriers (Eisen *et al*, 2005; Chang-Claude *et al*, 2007), the meta-analysis suggested that prophylactic oophorectomy offered a 53% reduction in risk of first primary breast cancer (95% CI 0.26–0.84).

In the current study, we did not observe a statistically significant reduction in contralateral breast cancer risk associated with the use of tamoxifen. This result differs from that of a case–control study by Gronwald *et al* (2006) that included 285 BRCA mutation carriers with contralateral breast cancer and 751 matched controls, in which tamoxifen use was associated with a contralateral breast cancer risk reduction of 50% for BRCA1 carriers and 58% for BRCA2 carriers. However, in none of the studies was tamoxifen associated with a risk reduction in women after oophorectomy. In the case–control study, 10% of the women had an oophorectomy, compared with 60% of the subjects in the current study.

The current study has several advantages over previous studies, including a large sample size, confirmation of cancers and all treatments with medical records, and the inclusion of deceased cases. All women with breast cancer in the families were identified and those who had been diagnosed with breast cancer from 1975 to 2008 at age 65 years or younger were eligible. Some women with breast cancer who had not received a test result were included, but women who had tested negative were excluded. We included untested and deceased women in the study to avoid the survivorship bias that would arise if only tested women were included. In many cases, testing was carried out several years after the diagnosis of breast cancer and restricting the study population to tested women would introduce survivorship bias. However, given the presence of a documented mutation in each family, the inclusion of untested women should not introduce significant misclassification bias. Within our cohort, 29 women with breast cancer were excluded because they had undergone genetic testing for the family BRCA mutation and were found not to carry the mutation. However, 711 women with breast cancer were found to have a BRCA mutation. Therefore, 740 women had genetic testing and 29 women were non-carriers. This suggests that <4% of women with breast cancer in families with a documented BRCA mutation do not have the family mutation. Therefore, of the 99 women included in this study, in which genetic testing had not been performed, we would expect that only 4 of the women (4%) would be non-carriers. This suggests that in 810 breast cancer cases included in this study, four of the cases (0.4%) may not have had a BRCA mutation. There are limitations as well. Ideally, we would conduct a prospective study based on unselected cases.

In summary, women with a BRCA mutation have a high risk of developing a contralateral breast cancer after the diagnosis of a first breast cancer. This risk is highest for women diagnosed with breast cancer under the age of 50 years and for those with multiple first-degree relatives affected with breast cancer. For women with intact ovaries, two or more first-degree relatives with breast cancer and who were diagnosed at age 49 years or below, the 15-year risk of contralateral breast cancer was 68%. For these high-risk women, both oophorectomy and contralateral mastectomy should be discussed as a component of the treatment plan to reduce the risk of second primary breast cancer and to prevent ovarian cancer.

## Figures and Tables

**Table 1:** Characteristics of study subjects

**Variable**	**Mean (range)**
Year of birth	1949.9 (1914–1981)
Age of diagnosis (years)	42.2 (21–65)
End of follow-up	2003.6 (1977–2009)
Follow-up (years)	11.5 (0.3–33.1)
	
**Variable**	***N*** (**%)**
*Age at diagnosis (in years)*	
<30	49 (6.1)
30–40	308 (38.0)
40–50	299 (36.9)
50+	154 (19.0)
	
*Mutation*	
BRCA1	498 (61.5)
BRCA2	300 (37.0)
Both	12 (1.5)
	
*Place of residence*	
Canada	500 (61.7)
United States	310 (38.3)
	
*Genetic testing*	
Yes	711 (87.8)
No	99 (12.2)
	
*Vital status*	
Living	638 (78.8)
Deceased	172 (21.5)
	
*Cause of death*	
Breast cancer	147
Second malignancy	10
Other	9
Unknown	6
	
*First-degree relative with breast cancer ⩽50 years*	
0	337 (42.8)
1	307 (39.1)
2+	144 (18.7)
Missing	20
Adopted	2
	
*Tumor size (cm)*	
0–2	500 (61.7)
2±5	291 (35.9)
Missing	19 (2.4)
	
*Grade*	
I	33 (4.1)
II	140 (17.3)
III	320 (39.6)
Unknown	319 (39.0)
	
*ER status*	
Positive	269 (33.2)
Negative	321 (39.6)
Equivocal	19 (2.4)
Unknown	201 (24.8)
	
*Lymph node status*	
Positive	287 (35.7)
Negative	518 (64.4)
Unknown	5
	
*Histology*	
Medullary	61 (7.5)
Ductal	682 (84.2)
Lobular	33 (4.1)
Other/unknown	34 (4.2)
**Variable**	***N*** (**%)**
*Surgery*	
Lumpectomy	396 (48.9)
Unilateral mastectomy	417 (41.1)
	
*Prophylactic surgery on contralateral breast*	
No	555 (68.5)
Yes	255 (31.5)
	
*Chemotherapy*	
No	260 (32.1)
Yes	533 (65.8)
Missing	17 (2.1)
	
*Tamoxifen*	
No	500 (61.8)
Yes	268 (33.1)
Missing	42 (5.1)
	
*Oophorectomy*	
No	303 (37.4)
Yes	489 (60.4)
Missing	18 (2.2)
	
*Timing of oophorectomy*	
Before diagnosis	40 (8.2)
After/at diagnosis	354 (72.4)
After/at contralateral BC	75 (15.3)
Missing	20 (4.0)
	
*Radiation therapy*	
No	373 (46.1)
Yes	424 (52.4)
Missing	12 (1.5)

**Table 2:** Cumulative risks of contralateral breast cancer

**Years from diagnosis**	**All subjects (%)**	**BRCA1 (%)**	**BRCA2 (%)**	**<50 years at diagnosis (%)**	**>50 years at diagnosis (%)**
5	13.1	13.7	12.0	14.2	8.6
10	22.0	23.8	18.7	23.9	14.7
15	33.8	36.1	28.5	37.6	16.8

**Table 3:** Relative risks of contralateral breast cancer associated with selected factors (all subjects)

**Variable**	**Univariate RR (95% CI) *P*-value**	**Multivariate^a^ RR (95% CI) *P*-value**	**Multivariate^b^ RR (95% CI) *P*-value**
*Mutation*			
BRCA1	1.0	1.0	1.0
BRCA2	0.86 (0.62–1.20) 0.38	0.88 (0.60–1.29) 0.51	0.86 (0.61–1.22) 0.41
			
*Age at diagnosis (years)*			
<40	1.0	1.0	1.0
40–49	0.78 (0.56–1.08) 0.14	0.92 (0.65–1.29) 0.61	0.91 (0.64–1.28) 0.58
⩾50	0.44 (0.26–0.73) 0.001	0.47 (0.27–0.82) 0.008	0.48 (0.28–0.82) 0.007
			
*Oophorectomy* ^c^			
No	1.0	1.0	1.0
Yes	0.49 (0.32–0.77) 0.002	0.48 (0.27–0.82) 0.002	0.53 (0.34–0.84) 0.007
			
*Chemotherapy*			
No	1.0	1.0	
Yes	0.87 (0.63–1.19) 0.37	0.99 (0.67–1.45) 0.94	
			
*Radiation therapy*			
No	1.0	1.0	
Yes	1.03 (0.75–1.42) 0.84	1.11 (0.79–1.55) 0.56	
			
*Tamoxifen*			
No	1.0	1.0	1.0
Yes	0.64 (0.44–0.93) 0.02	0.72 (0.47–1.12) 0.14	0.74 (0.49–1.11) 0.14
			
*ER status*			
Negative	1.0	1.0	
Positive	0.80 (0.54–1.19) 0.26	1.02 (0.64–1.62) 0.95	
			
*Grade*			
I/II	1.0	1.0	
III	1.00 (0.62–1.61) 0.99	0.84 (0.50–1.41) 0.51	
			
*Nodal status*			
Negative	1.0	1.0	
Positive	0.74 (0.52–1.03) 0.08	0.76 (0.51–1.12) 0.16	
			
*First-degree relative with breast cancer ⩽50*			
0	1.0	1.0	1.0
1	1.27 (0.89–1.81) 0.20	1.15 (0.80–1.67) 0.45	1.19 (0.83–1.71) 0.36
2+	1.70 (1.13–2.54) 0.01	1.86 (9.22–2.83) 0.004	1.79 (1.18–2.71) 0.006
Trend	1.30 (1.06–1.59) 0.01	1.34 (1.08–1.66) 0.008	1.33 (1.08–1.64) 0.008

a Multivariate estimates are adjusted by all variables.

b Multivariate estimates are adjusted by four variables

c Time dependent variable in the regression of Cox's proportional hazards model.

**Table 4:** Relative risks of contralateral breast cancer associated with selected factors by age at diagnosis

**Variable**	**Univariate RR (95% CI) *P*-value**	**Multivariate RR (95% CI) *P*-value** a	**Multivariate RR (95% CI) *P*-value** b
*Breast cancer diagnosis under age 50*			
*Mutation*			
BRCA1	1.0	1.0	1.0
BRCA2	0.92 (0.65–1.31) 0.64	0.90 (0.59–1.35) 0.59	0.88 (0.61–1.27) 0.50
			
*Age at diagnosis (in years)*			
<40	1.0	1.0	1.0
40–49	0.78 (0.56–1.08) 0.13	0.93 (0.66–1.31) 0.68	0.92 (0.65–1.30) 0.63
			
*Oophorectomy*^c^			
No	1.0	1.0	1.0
Yes	0.45 (0.27–0.74) 0.002	0.39 (0.23–0.67) 0.0006	0.45 (0.26–0.75) 0.002
			
*Chemotherapy*			
No	1.0	1.0	
Yes	0.83 (0.59–1.17) 0.29	1.08 (0.72–1.63) 0.71	
			
*Radiation therapy*			
No	1.0	1.0	
Yes	1.03 (0.73–1.45) 0.86	1.11 (0.78–1.60) 0.56	
			
*Tamoxifen*			
No	1.0	1.0	1.0
Yes	0.69 (0.45–1.04) 0.08	0.69 (0.42–1.11) 0.12	0.73 (0.46–1.14) 0.16
			
*ER status*			
Negative	1.0	1.0	
Positive	0.86 (0.57–1.32) 0.50	1.09 (0.67–1.78) 0.73	
			
*Grade*			
I/II	1.0	1.0	
III	0.86 (0.51–1.43) 0.56	0.75 (0.43–1.31) 0.32	
			
*Nodal status*			
Negative	1.0	1.0	
Positive	0.66 (0.46–0.95) 0.03	0.66 (0.44–1.00) 0.05	
			
*First-degree relative with breast cancer ⩽50*			
0	1.0	1.0	1.0
1	1.18 (0.80–1.73) 0.40	1.08 (0.73–1.61) 0.72	1.09 (0.74–1.62) 0.65
2+	1.95 (1.28–2.97) 0.002	2.14 (1.36–3.33) 0.0008	2.01 (1.31–3.09) 0.002
Trend	1.38 (1.11–1.71) 0.004	1.42 (1.12–1.78) 0.003	1.39 (1.11–1.74) 0.004
			
*Breast cancer diagnosis 50 years or older*			
*Mutation*			
BRCA1	1.0	1.0	1.0
BRCA2	0.91 (0.36–2.32) 0.85	0.59 (0.17–1.98) 0.39	0.95 (0.33–2.72) 0.92
			
*Oophorectomy*^c^			
No	1.0	1.0	1.0
Yes	1.03 (0.38–2.81) 0.95	0.90 (0.30–2.64) 0.84	1.02 (0.36–2.97) 0.97
			
*Chemotherapy*			
No	1.0	1.0	
Yes	0.64 (0.24–1.74) 0.38	0.32 (0.07–1.56) 0.16	
			
*Radiation therapy*			
No	1.0	1.0	
Yes	0.71 (0.28–1.81) 0.47	0.57 (0.18–1.84) 0.35	
			
*Tamoxifen*			
No	1.0	1.0	1.0
Yes	0.79 (0.29–2.12) 0.63	1.13 (0.29–4.33) 0.86	0.64 (0.22–1.89) 0.42
			
*ER status*			
Negative	1.0	1.0	
Positive	0.91 (0.28–2.99) 0.87	0.85 (0.17–4.36) 0.84	
			
*Grade*			
I/II	1.0	1.0	
III	2.20 (0.55–8.71) 0.26	3.33 (0.69–1.61) 0.13	
*Nodal status*			
Negative	1.0	1.0	
Positive	1.10 (0.39–3.09) 0.86	2.00 (0.45–8.91) 0.36	
			
*First-degree relative with breast cancer ⩽50*			
0	1.0	1.0	1.0
1	1.97 (0.70–5.50) 0.20	1.34 (0.40–4.56) 0.64	1.88 (0.64–5.53) 0.25
2+	0.72 (0.14–3.57) 0.68	0.49 (0.08–3.08) 0.45	0.91 (0.18–4.64) 0.91
Trend	1.02 (0.55–1.87) 0.96	0.79 (0.36–1.74) 0.56	1.10 (0.57–2.12) 0.78

Abbreviations: CI=confidence interval; ER=oestrogen receptor; RR=relative risk.

a Multivariate estimates are adjusted by all variables;

b Multivariate estimates are adjusted by only four variables;

c Time-dependent variable in the regression of Cox's proportional hazards model.

**Table 5:** Relative risks of contralateral breast cancer associated with selected factors by mutation

**Variable**	**Univariate RR (95% CI) *P*-value**	**Multivariate RR (95% CI) *P*-value** ^**a**^	**Multivariate RR (95% CI) *P*-value^b^**
*BRCA1*			
*Age at diagnosis*			
<40	1.0	1.0	1.0
40–49	0.62 (0.410.96) 0.03	0.72 (0.47–1.12) 0.14	0.71 (0.46–1.10) 0.13
⩾50	0.43 (0.22–0.84) 0.21	0.54 (0.27–1.11) 0.09	0.53 (0.27–1.06) 0.07
			
*Oophorectomy*^c^			
No	1.0	1.0	1.0
Yes	0.43 (0.25–0.74) 0.002	0.48 (0.27–0.84) 0.01	0.52 (0.30–0.91) 0.02
			
*Chemotherapy*			
No	1.0	1.0	
Yes	0.89 (0.59–1.33) 0.57	1.09 (0.68–1.74) 0.72	
			
*Radiation therapy*			
No	1.0	1.0	
Yes	1.08 (0.72–1.61) 0.71	1.13 (0.74–1.72) 0.56	
			
*Tamoxifen*			
No	1.0	1.0	1.0
Yes	0.55 (0.31–0.94) 0.03	0.61 (0.33–1.13) 0.12	0.66 (0.37–1.15) 0.14
			
*ER status*			
Negative	1.0	1.0	
Positive	0.57 (0.31–1.07) 0.08	0.81 (0.42–1.58) 0.54	
			
*Grade*			
I/II	1.0	1.0	
III	0.87 (0.47–1.63) 0.67	0.66 (0.34–1.28) 0.22	
			
*Nodal status*			
Negative	1.0	1.0	
Positive	0.80 (0.51–1.25) 0.32	0.76 (0.47–1.25) 0.28	
			
*First-degree relative with breast cancer ⩽50*			
0	1.0	1.0	1.0
1	1.42 (0.91–2.23) 0.13	1.34 (0.84–2.13) 0.22	1.35 (0.86–2.13) 0.19
2+	1.80 (1.09–3.00) 0.02	1.97 (1.17–3.31) 0.01	1.94 (1.16–3.23) 0.01
Trend	1.35 (1.05–1.73) 0.02	1.40 (1.08–1.81) 0.01	1.39 (1.08–1.80) 0.01
			
*BRCA2*			
*Age at diagnosis (in years)*			
<40	1.0	1.0	1.0
40–49	1.20 (0.68–2.14) 0.53	1.73 (0.91–3.29) 0.09	1.41 (0.76–2.60) 0.28
⩾50	0.56 (0.25–1.27) 0.16	0.46 (0.18–1.16) 0.10	0.48 (0.19–1.18) 0.11
			
*Oophorectomy*^c^			
No	1.0	1.0	1.0
Yes	0.58 (0.26–1.32) 0.19	0.49 (0.21–1.17) 0.11	0.51 (0.22–1.19) 0.12
			
*Chemotherapy*			
No	1.0	1.0	
Yes	0.77 (0.44–1.27) 0.28	0.94 (0.44–2.05) 0.88	
			
*Radiation therapy*			
No	1.0	1.0	
Yes	0.87 (0.51–1.50) 0.62	0.76 (0.41–1.39) 0.37	
			
*Tamoxifen*			
No	1.0	1.0	1.0
Yes	0.88 (0.49–1.58) 0.67	0.86 (0.43–1.73) 0.68	0.98 (0.51–1.86) 0.95
			
*ER status*			
Negative	1.0	1.0	
Positive	2.62 (0.92–7.50) 0.07	3.30 (1.06–10.3) 0.04	
			
*Grade*			
I/II	1.0	1.0	
III	1.26 (0.56–2.84) 0.59	1.62 (0.68–3.84) 0.28	
			
*Nodal status*			
Negative	1.0	1.0	
Positive	0.65 (0.38–1.14) 0.13	0.56 (0.26–1.19) 0.13	
			
*First-degree relative with breast cancer ⩽50*			
0	1.0	1.0	1.0
1	0.95 (0.52–1.75) 0.87	0.77 (0.40–1.46) 0.42	0.83 (0.44–1.57) 0.56
2+	1.58 (0.80–3.13) 0.91	1.85 (0.86–3.98) 0.11	1.52 (0.74–3.13) 0.25
Trend	1.22 (0.85–1.74) 0.28	1.22 (0.81–1.83) 0.34	1.18 (0.81–1.73) 0.39

Abbreviations: CI=confidence interval; ER=oestrogen receptor; RR=relative risk.

a Multivariate estimates are adjusted by all variables;

b Multivariate estimates are adjusted by only four variables;

c Time-dependent variable in the regression of Cox's proportional hazards model.
